# Does the insulin pump improve satisfaction and glycaemic control in Moroccan patients with type 1 diabetes?

**DOI:** 10.1900/RDS.2023.19.8

**Published:** 2023-03-31

**Authors:** Najoua Messaoudi, Abir Tahri, Imane Assarar, Nisrine Bouichrat, Nada Derkaoui, Mohammed Amine Bouazzaoui, Siham Rouf, Naima Abda, Hanane Latrech

**Affiliations:** 1 Department of Endocrinology-Diabetology and Nutrition, Mohammed VI University Hospital Center, Faculty of Medicine and Pharmacy, University of Mohammed 1st, Oujda, Morocco,; 2 Laboratory of Epidemiology, Clinical Research and Public Health, Faculty of Medicine and Pharmacy, University of Mohammed first, Oujda, Morocco.

**Keywords:** type 1 diabetes, insulin-pump, satisfaction, glycaemic-control

## Abstract

**Introduction:**

Insulin pump therapy is recommended more and more to achieve and maintain optimal glycaemic control in patients with type 1 diabetes mellitus. The objective of our study was to evaluate the satisfaction of patients using insulin pump therapy and to determine its effectiveness in improving metabolic control in type 1 diabetic patients.

**Patients-Methods:**

This is a retrospective, descriptive and analytical study including 20 type 1 diabetic patients treated by insulin pump, between 2017 and 2021. All patients received a clinical evaluation, analysis of capillary blood glucose monitoring and a dosage of HbA1c at the time of the start of insulin pump and during the evolution. Insulin pump satisfaction was assessed using the Diabetes Treatment Satisfaction Questionnaire (DTSQ). Statistical analysis was performed by SPSS version-21.

**Results:**

The mean age of the patients was 16,8 ± 8,1 years with a sex ratio (M/F) of 0,42. Thirty per-cent were children. The mean duration of diabetes was 5,8 ± 4,8 years. Seventy-five per-cent of patients practiced functional insulin therapy. The indications for insulin pump treatment were mainly hypoglycaemia and instable diabetes. During follow-up, we found a statistically significant decrease in insulin requirements, improvement in mean HbA1c and maintenance of glycaemic control during follow-up, with a marked reduction in the number of hypoglycaemia events per week. The overall satisfaction score was calculated at 34,6 ± 2,5 out of 36 with a decrease in the score for perception of hyperglycaemia or hypoglycaemia.

**Conclusion:**

Insulin pump therapy appears to be reliable and effective when used appropriately, combined with appropriate therapeutic education and glycaemic monitoring to maintain long-term glycaemic control and improved quality of life.

## 1. Introduction

Since its introduction in the 1970s, insulin pump therapy has generated a particular interest, representing an important advance in the management of patients with type 1 diabetes [1]. When comparing insulin pump therapy with the conventional or multi-injection (Basal-Bolus) regimen, it essentially allows for greater satisfaction, a more flexible lifestyle, an optimal glycaemic control with an improvement in the frequency of hypoglycaemic episodes, a reduction in HbA1c levels and glycaemic variability, as well as avoiding chronic complications such as diabetic retinopathy and nephropathy [2-4].

Insulin pump therapy is becoming increasingly recommended for patients with type 1 diabetes. However, metabolic control on insulin pumps has been widely studied, in contrast to quality of life and satisfaction, which has unfortunately only been studied in a limited number of studies [3-5]. Moreover, the benefits of insulin pump therapy on glycaemic control have never been studied in Moroccan population. In fact, in our country, it seems difficult to obtain an insulin pump due to the high and permanent cost of the equipment, the lack of coverage and reimbursement by all medical insurance companies, which makes the number of patients treated by insulin pump very limited. No matter the difficulties, our study is the first monocentric and largest series in our country reporting the impact of the insulin pump in a developing country on glycaemic control and satisfaction.

The main objective of our study was to determine the effectiveness of insulin pump therapy in type 1 diabetic patients in terms of their satisfaction as assessed by the DTSQ (Diabetes Treatment Satisfaction Questionnaire). The secondary objectives were to evaluate the metabolic control of these patients in terms of HbA1c, the frequency of hypoglycaemia per week, and the effect on body mass index (BMI) during the follow-up; at the first setting up of insulin pump (M0), 3 months (M3), 6 months (M6), 1 year (M12), 2 years (M24), 3 years (M36) and 4 years (M48) of follow-up.

## 2. Patients and Methods

### 2.1 Study Design

This is a retrospective, descriptive and analytical cross-sectional study, conducted over a period of 4 years (from 2017 to 2021) in the Department of EndocrinologyDiabetology and Nutrition of Mohammed VI University Hospital Center, in the eastern region of Morocco. All enrolled patients had complete medical records and have given their oral consent to participate in the study.

### 2.2 Study population

Twenty patients with type 1 diabetes, who have been using an insulin pump for at least 3 months were enrolled. We excluded from this study patients who used the pump for less than 3 months, type 2 diabetes patients using an insulin pump and patients with incomplete medical records.

### 2.3 Study protocol

**Figure 1.**
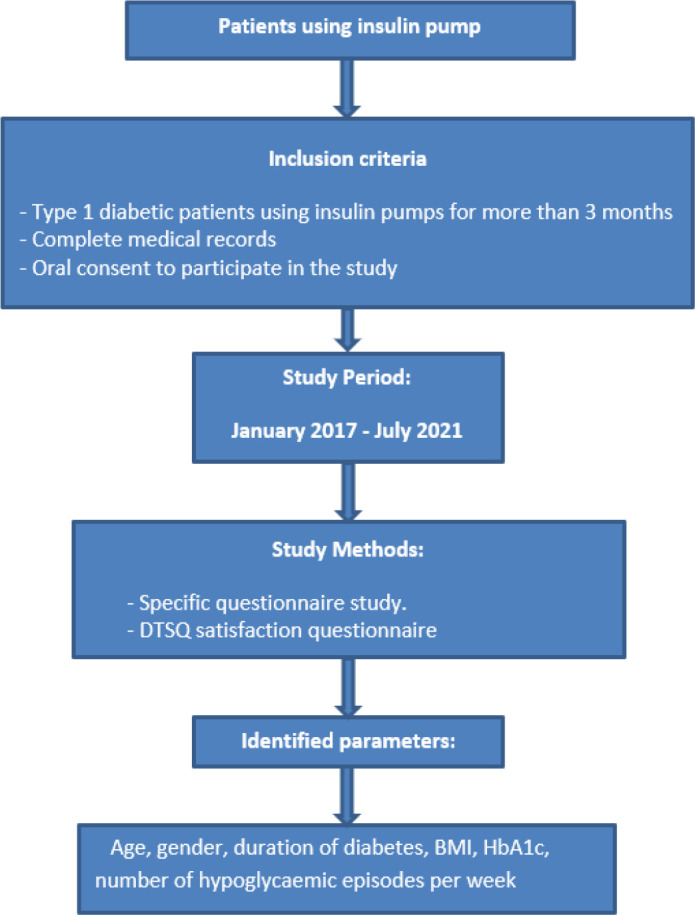
The general plan showing the design of the study

After inclusion, patient data were collected from medical records using a specific questionnaire prepared for this study and covering socio-demographic parameters, clinical examination data, and diabetes-related data (Figure 1).

Satisfaction was assessed using the DTSQ, which was first developed in 1990 by psychologist Clare Bradley, to measure patients’ satisfaction with their diabetes treatment [6]. It was translated into several languages, including Arabic. It is divided into 2 parts with 8 questions in total. The first part evaluates the satisfaction with the treatment and consists of 6 questions (Q1, 4, 5, 6, 7 and 8), including: ‘satisfaction with current treatment’, ‘flexibility’, ‘convenience of the treatment’, ‘satisfaction with own understanding of their diabetes’, ‘how likely to recommend their present treatment’ and ‘how satisfied to continue with their present treatment’ respectively. The second part of the questionnaire assesses the burden of hypo- and hyperglycaemia, consisting of 2 questions (Q 2 and 3). The response to these questions is scored on a scale ranging from very dissatisfied to very satisfied for questions (Q1, 4, 5, 6, 7, 8) and from 0 (never) to 6 (most of the time) for questions 2 and 3.

In our study, we used the version translated into Arabic for Morocco, knowing that this questionnaire has never been validated before for our country. For this matter, our medical team contacted the Health Psychology Research group, and a validation process was followed for several months. This process includes a linguistic validation followed by the signature of the operating license and the cognitive debriefing process followed by correction steps. In the end, we obtained the version validated by the Health Psychology Research group of the DTSQs in Arabic for Morocco.

### 2.4 Statistical analysis

All analyses were performed with SPSS (Statistical Package for the Social Sciences), version 21 (IBM, Armonk, NY). The different data were distributed and presented as means. The comparison of results, including HbA1c, hypoglycaemic episodes, and BMI was performed using SPSS version 21.0.

## 3. Results

### 3.1 Characteristics of the population

Twenty type 1 diabetic patients were enrolled in the study, 14 were female and 6 were male, with a sex ratio M/F (male/female) of 0.42. The mean age at inclusion was 16.8 ± 8.1 years, with 45% adults, 25% adolescents and 30% children. The mean duration of diabetes was 5.8 ± 4.8 years. Thirty percent of the patients had a family history of type 1 diabetes, 50% had a family history of type 2 diabetes.

The mean age at initiation of insulin pump therapy was 15.8 ± 7.9 years. The indications for insulin pump therapy were hypoglycaemia, brittle diabetes, glycaemic imbalance and dawn phenomenon in 45%, 30%, 15% and 10% of cases respectively. All patients were treated with the basal-bolus regimen before insulin pump therapy, with an average number of injections per day of 4.3 ± 0.5 injections per day (corresponding to intensified diabetes treatment). Seventy-five percent of patients were on functional insulin therapy (FIT).

### 3.2 The results of the study

#### 3.2.1 Insulin requirements

Insulin requirements (rapid-acting insulin + basal insulin) before initiation of the insulin pump were estimated at 40.5 ± 24.5Ui per day, i.e., 0.82 ± 0.34Ui per kg, with higher requirements in adolescents (60.6 ± 22Ui per day). For basal insulin, the requirement before initiation of the insulin pump was calculated to be 15 ± 9Ui, i.e., 0.30 ± 0.12Ui per Kg. After insulin pump use, we found a statistically significant decrease in insulin requirements during the follow-up (P = 0.04) (Table 1).

**Table 1.** Evolution of basal insulin requirements during follow-up on insulin pump therapy in Ui/Jr

	Before starting the Insulin Pump	At the start of insulin pump (M0)	M3	M6	M12	M24
The average basal insulin requirement (Ui/day)	15 ± 9	11,8 ± 7,5	11,1 ± 6.9	10,1 ± 6	13,62 ± 9	12 ± 9,5
In children (Ui/day)	6,2 ± 4.7	4,7 ± 3,3	5,2 ± 3,2	5,3 ± 3	3,67 ± 0,3	3,68 ± 0,5
Adolescents (Ui/day)	21,6 ± 8,2	16,9 ± 8,8	15,2 ±7,1	14,7 ± 4,3	19,6 ± 8,2	19 ± 9,7
Adults (Ui/day)	17,5 ± 7	14 ± 5,2	11,3 ± 6,6	11 ± 6.4	14,5 ± 6,3	14,7 ± 6

Data is expressed in n (%) or mean (SD). HbA1c = hemoglobin A1c. The comparisons were analyzed using t-student test and chi-square test. A value of p&lt;0.05 indicated significance.

#### 3.2.2 Metabolic control

The average BMI at the initiation of the insulin pump therapy was 20.7 ± 3.5 kg/m^2^. Its value decreased to 19.9 ± 1 kg/m^2^ after 6 months of use and remained stable afterwards. In the paediatric population, a regular weight growth was noted.

Considering metabolic control, assessed by HbA1c, we found a statistically significant improvement at M6 of follow-up (p = 0.02). Afterwards, HbA1c was stable, apart from a 1% elevation at M 36 of follow-up in children, which coincided with the summer season. (Table 2)

**Table 2.** Assessment of HbA1c during follow-up on insulin pumps

	M0	M3	M6	M12	M24	M36	M48
Average HbA1c (%)	7,5 ± 1	7,3 ± 0,7	7 ± 0,7	7,4 ± 0,5	7,1 ± 0,8	7,7 ± 0,6	7,3 ± 0,1
In children	7 ± 0,9	7 ± 0,7	7 ± 0,3	7,1 ± 0,5	6,2 ± 0,9	7,2 ± 0,1	7 ± 0,3
Adolescents	8,1 ± 0,5	8 ± 0,4	7,7 ± 0,4	7,7 ± 0,6	7,6 ± 0,1	8 ± 0,6	7,5 ± 0,5
Adults	7,5 ± 1,3	7 ± 0,7	6,7 ± 0,8	7,2 ± 0,1	7,5 ± 0,42	8 ± 0,7	7,3 ± 0,5

Data is expressed in n (%) or mean (SD). HbA1c = hemoglobin A1c. The comparisons were analyzed using t-student test and chi-square test. A value of p&lt; 0.05 indicated significance

In terms of hypoglycaemic events per week, the mean number of hypoglycaemias per week before insulin pump therapy was 4.8 ± 3 episodes per week, with 6.5 ± 3.2 episodes per week in children. At M3, there was a statistically significant reduction in the number of hypoglycaemias to 1.82 ± 1 (p<0.05), at M6 the number of hypoglycaemias further decreased to 1 episode per week. For adolescents, at M12, there were no episodes of hypoglycaemia during insulin pump treatment.

#### 3.3.3 Satisfaction

In terms of satisfaction, for the adult patients who had answered the DTSQ (45% of the study population), the global satisfaction score for Q1 ,4 ,5 ,6 ,7 and 8 (Satisfaction with insulin pump treatment) was calculated at 34.6 ± 2.5 out of 36. We noted an almost complete response of 5.93 ± 0.2 out of 6 for questions 7 and 8 judging the ‘recommendation of treatment to others’ and the ‘willingness to continue the same treatment’. Despite the small sample of the study, we found that the satisfaction score tended to decrease with the duration of diabetes.

For questions 2 and 3 of the DTSQ, reporting the impression of the sensation of hyperglycaemia and hypoglycaemia, the score was rated at 3.3 ± 1.3 out of 6 and 2.7 ± 1.7 out of 6 respectively (Table 3).

**Table 3.** Answers to the DTSQ Satisfaction Questionnaire

Question	Score
Q1: satisfaction with current treatment	5,6 ± 0,3
Q2: charge of hyperglycaemia	3,2 ± 1,3
Q3: charge of hypoglycaemia	2,8 ± 1,7
Q4: flexibility	5,7 ± 0,5
Q5: Commodity	5,5 ± 1
Q6: the understanding of diabetes	5,8 ± 0,4
Q7: the recommendation of treatment to others	5,9 ± 0,3
Q8: the willingness to continue	5,9 ± 0,3
The Global Score (Q1, 4, 5, 6, 7, et 8)	34,6 ± 2,5

The DTSQ has been validated in 2 other versions, one for parents of diabetic children and one for adolescents, but it is not yet validated in Arabic.

#### 3.3.4 Autoimmune and degenerative status assessment

In terms of other autoimmune diseases, one patient was followed-up for coeliac disease with autoimmune thyroiditis, and isolated autoimmune thyroiditis was noted in 2 other patients. For degenerative complications, a minimal diabetic retinopathy was noted in one patient, and a positive microalbuminuria in another patient.

## 4. Discussion

Type 1 diabetes is a heterogeneous disease, characterized by a destruction of the beta-pancreatic cells, mostly of autoimmune origin, leading to an absolute insulin deficiency. Type 1 diabetes accounts for 5-10% of all types of diabetes worldwide, affecting mostly children and adolescents [7]. The management of this type of diabetes is currently based on insulin therapy using a multi-injection regimen or continuous subcutaneous insulin infusion via an external insulin pump [8].

Since its introduction in 1970, the insulin pump has aroused particular interest. It was first commercialized in 1983, and since then the production of pumps went through a rapid progress. Currently, it is considered as the first-line treatment for patients with type 1 diabetes mellitus. In the United States, 64% of type 1 diabetic patients are on insulin pump, and the rate of use in Europe varies from 70% to 93% [9]. In children, this therapy was initiated the 2000s, not until the results of the DCCT study (the Diabetes Control and Complications Trial) were unveiled. The DCCT study demonstrated the value of insulin pump therapy in maintaining glycaemic control and preventing microvascular complications in children and adults with diabetes.

Various other studies have examined the contribution of the insulin pump to glycaemic control, the reduction of insulin requirements and the reduction in terms of hypoglycaemia frequency. In children and adolescents, JA. Ahern et al. [10] reported a significant reduction in insulin requirements and HbA1c levels with maintained glycaemic control over a mean follow-up of 32 months. This has been further demonstrated in multiple observational studies, which reported a significant improvement in glycaemic control with a decrease in HbA1c of 0.3 to 1% during follow-up [11, 12, 13] (Table 4). In our series, HbA1c in adolescents decreased from 8.1 ± 0.5% to 7.7 ± 0.6% after one year. In adults, Didangelos T. et al. [14] reported the results of a meta-analysis of 52 studies including 1547 patients, showing a significant reduction in HbA1c (p=0.039), although this reduction seems to be significant only when the pump is used for at least one year. In our series, a reduction in HbA1c was observed in adults since the third month, with an increase of 0.5% at M36 of follow-up.

**Table 4.** A table summarizing the metabolic benefits of the insulin pump

	Age range	Age (years)	Insulin requirement (Ui/ Kg/Day) (Total insulin or basal insulin)		HbA1c (%)		Frequency of hypoglycaemia (Per year or per week) (severe hypoglycaemia)	
			Pre-pump	12 months post-pump	Pre-pump	12 months post-pump	Pre-pump	12 months post-pump
JA. Ahern et al. (N= 161 Patients)[10]	Pre-school age children (1-6 years)	3,9 ± 1,1	0,7 ± 0,2	0,8 ± 0,2	7,1 ± 0,9	6,5 ± 0,7	11 ± 0,5 per year	5 ± 0,2 per year
	School age children (7-11 years)	9,1 ± 0,9	1 ± 0,6	0,9 ± 0,3	7,9 ± 1	7,3 ± 1,1	25 ± 0,3 per year	17 ± 0,2 per year
	Teenagers (12-18 years old)	14,7 ±1,4	1,3 ± 0,5	0,9 ± 0,5	8,1 ± 1,5	7,4 ± 1,2	20 ± 0,3 per year	16 ± 0,3 per year
Cohen et al. [11]	Children and teenagers	14,2			8,5 ± 1,4 à 8,6 ± 0,5	8,6 ± 0,8 à 8,1± 1,3		
Weinzimer et al. [12]	Children	4,5 (1.4-6.9)			7,4 ± 1	7 ± 0,9		
Nuboer et al. [13]	Children	12 (4-16)			7,9 ± 0.6 à 7,7±0,9	7,6 ±0,6 à 7,53± 0,67		
Didangelos T. et al. [14]	Adults				9,4 ± 0,2%	8,9 ± 0,1%		
Our Series	Children (1-11 years)	6,6 ± 2,6	Basal Insulin per Kg per day		7 ± 0,9	6,2 ± 0,9	Episode of hypoglycaemia per week	
			0,25 ± 0,08	0,22 ± 0,06			6,5 ± 3,2	1,5 ± 0,7
	Adolescents (12-18 years old)	17,8 ± 6	0,4 ± 0,1	0,33 ± 0,2	8,1 ± 0,5	7,7 ± 0,6	4,4 ± 3,5	0,7
	Adults	23 ± 8,1	0,3 ± 0,12	0,23 ± 0,08	7,5 ± 1,3	7,2 ± 0,1	2	3,88 ± 2

In the previous studies, as well as in ours, it has been shown that with intensified treatment of diabetes, including subcutaneous insulin pump therapy, episodes of severe hypoglycaemia tend to decrease with lowering of HbA1c, contrary to what was reported by the DCCT study, which indicated that the risk of severe hypoglycaemia increased substantially when HbA1c levels were decreased [10].

Insulin requirements vary according to age and pubertal stage, with increased requirements in adolescents. In our series, despite the small sample population, we found a decrease in insulin requirements during the evolution with insulin pump compared to the requirements with multi-injection regimen, which has also been proven in several studies [15].

The evaluation of the effectiveness of insulin pump therapy should not be based on the improvement of glycaemic control alone, but should also include satisfaction with the therapy. Indeed, an individual’s perception of his or her health is one of the most significant qualitative indicators of health studied nowadays. There are a number of tools to assess patients’ health-related life measures and their satisfaction with the care received, the DTSQ is one of these instruments, aimed at assessing satisfaction with the care given to patients with diabetes mellitus [16].

The DTSQ was developed by Clare Bradley, an English health psychologist, in the 1990’s with the objective of evaluating patient satisfaction with their diabetes treatment. It consists of 2 parts, divided into 8 questions. Treatment satisfaction is assessed as the sum of the scores of the six questions on the first part (total score 36), a higher score indicating greater treatment satisfaction [16].

Studies reporting the assessment of satisfaction by the DTSQ in type 1 diabetic patients are few.

Maiorino. M.I et al. [17] demonstrated in their paper published in 2013, a significant increase in the DTSQ score associated with a significant decrease in the score related to the perception of hyperglycaemia of hypoglycaemia in type 1 diabetic patients treated with insulin pump compared to those under multi-injection regimen. On the other hand, in a large case-control study published in 2008 involving adults with type 1 diabetes, insulin pump treatment was associated with a significantly higher DTSQ score compared to those treated with a multi-injection regimen [18]. In our series, despite the small population, we found that the satisfaction score was high, rated at 34.6 ± 2.5 out of 36.

The limitations of our study consist in the small number of patients in our sample. This can be explained by the high and permanent cost of the equipment, the lack of coverage and reimbursement by all medical insurance companies, which limits the number of patients who have the access to use an insulin pump in our developing country.

### 4.1 Conclusion

After 50 years of history, the insulin pump has demonstrated its effectiveness in improving glycaemic control, reducing glycaemic fluctuations, obtaining better flexibility and autonomy, hence a better quality of life. In order to achieve these objectives, the insulin pump must be used in an appropriate way, which requires patient education and continuous education of healthcare providers. In our country, as previously mentioned; it seems difficult to obtain an insulin pump due to the high and permanent cost of the equipment, the lack of coverage and reimbursement by all medical insurance companies, which makes the number of patients treated by insulin pump very limited.

## Abbreviations

BMI: Body mass index

DTSQ: Diabetes Treatment Satisfaction Questionnaire

DCCT: Diabetes Control and Complications Trial

HbA1c: Glycated haemoglobin

FIT: Functional insulin therapy
